# Cognitive Impairment in Patients with Pseudotumor Cerebri Syndrome

**DOI:** 10.3233/BEN-2011-0325

**Published:** 2011-05-23

**Authors:** Siddharth Kharkar, Robert hernandez, Sachin Batra, Philippe Metellus, Argye Hillis, Michael A. Williams, Daniele Rigamonti

**Affiliations:** ^1^Department of NeurologyJohns Hopkins School of MedicineBaltimoreMDUSA; ^2^Department of NeurosurgeryJohns Hopkins School of MedicineBaltimoreMDUSA; ^3^Adult Hydrocephalus ProgramJohns Hopkins HospitalBaltimoreMDUSA

**Keywords:** Cognition, impairment, morbidity, pseudotumor cerebri syndrome

## Abstract

*Introduction:* Patients with Pseudotumor Cerebri Syndrome (PTCS) may complain of difficulty in thinking or concentrating; however there has been little formal cognitive evaluation in this population.

*Objective:* To evaluate the characteristics and nature of cognitive impairment in patients with PTCS.

*Methods:* We retrospectively reviewed records of 10 patients diagnosed with PTCS who were cognitively tested at presentation. In each cognitive test, “Borderline deficit” (BD) was defined as a score more than 1 standard deviation (SD) below and “Definite Deficit” (DD) as a score more than 2 SD below the mean for age, sex and education. In each cognitive domain, impairment was defined as a single test score more than 2 SD below the mean, or scores of more than 1 SD below the mean for age, sex and education in > 50% of tests.

*Results:* Mean age of patients was 43.4 ± 13.5 years. 8/10(80%) patients were female. 3/10(30%) had papilledema; 3/10(30%) had significant cerebral venous outflow obstruction. Impairment was most commonly seen and was most severe in the WMS logical memory I (BD44%, DD44%), WMS logical memory II (BD37.5%, DD50%), RAVLT delayed recall (BD30%, DD40%) and RAVLT retention(BD40%, DD30%) tests. Evaluation of cognitive domains revealed impairment in memory and learning (80%), executive function (10%), visuospatial skills (30%), and language (30%).

*Conclusion:* Our results indicate that patients with PTCS can have significant cognitive impairment, particularly in learning and memory. The prevalence needs to be studied in a larger cohort. The relationship of cognitive impairment with chronically elevated intracranial pressures and its role in contributing to patient morbidity needs to be investigated further.

## 1. Introduction

Pseudotumor Cerebri Syndrome (PTCS) has been defined as the presence of increased Intracranial Pressure (ICP) without clinical, laboratory or radiological evidence of intracranial pathology on conventional imaging [1,2]. Symptoms of PTCS include headache, diplopia, visual blurring, nausea and vomiting.

There is very little published data to either prove or disprove the commonly held belief that patients with PTCS do not have significant cognitive impairment. Very few studies on this subject have involved cognitive testing using specialized tests [3–5]. This is of particular importance because the Mini mental status exam (MMSE) – which is commonly used by many clinicians as a screening tool – is very insensitive at the commonly utilized cut-off of 24 points [6] and the negative predictive value drops drastically with a drop in the prevalence of dementia in the study population [7].

We retrospectively reviewed the records of all PTCS patients who had undergone cognitive testing. This study was conducted with the aim of determining if cognitive deficit exist in PTCS and their nature.

## 2. Methods

### 2.1. Patient selection

Criteria used for the diagnosis of PTCS at our center have been previously described [8] (24). Briefly,

ISSN 0953-4180/11/$27.50 © 2011 – IOS Press and the authors. All rights reserved

Table 1
Cognitive symptoms, mood disorders and problems with normal functioning

| Symptom | Number (%) of patients reporting the symptom ($n = 10$) |
|---|---|
| 1. Problems with thinking or memory | 5 (50%) |
| a. Recent memory | 5 (50%) |
| b. Long term memory | 2 (20%) |
| c. Problems balancing bills or organizing daily schedule | 3 (30%) |
| 2. Mood disorders | |
| a. Depression | 2 (18%) |
| b. Anxiety | 1 (9%) |
| c. Both | 0 (0%) |
| 3. Problems with normal functioning | |
| a. Need assistance for self-care or for daily activities including dressing, using the toilet, bathing or eating | 2 (20%) |
| b. Problems with normal social functioning | 3 (30%) |
| c. Problems with work/occupation | 5 (50%) |

these are: 1) symptomatology consistent with generalized intracranial hypertension or noted papilledema; 2) ICP $\geqslant$ 25 cm H2O as measured in the lateral decubitus position; 3) normal CSF composition; 4) absence of hydrocephalus or a mass, structural, or vascular lesion on MR imaging; and 5) no other identifiable systemic cause of intracranial hypertension. All patients with suspected PTCS were asked to answer a detailed questionnaire before being clinically evaluated. Since some patients evaluated for PTCS at our center complained of problems with cognition, we referred these patients for cognitive testing. In the latter part of this series, patients who did not complain of cognitive difficulties were also tested cognitively to look for the presence of impairment. This group of patients was therefore a heterogeneous group of patients with and without cognitive complaints, as depicted in “Table 1”. Ophthalmoscopy was performed on all individuals to check for the presence or absence of papilledema.

### 2.2. *Treatment details*

At the time of evaluation, 2/10 patients (20%) patients were on treatment with diuretics only, while 2/10 (20%) patients were taking both diuretics and acetazolamide. 2 patients had a Cerebrospinal Fluid (CSF) shunt at the time of evaluation. None of the other patients had a history of being previously shunted for their condition.

7/10 patients (70%) did not have papilledema. Significant cerebral venous outflow obstruction was confirmed in 3 /10 (30%) patients.

### 2.3. *Cognitive testing*

All patients were assessed using the Mini Mental Status examination (MMSE) and a battery of cognitive tests. The cognitive battery included the following tests: (1) Revised Wechsler Memory Scale (WMS-R) logical memory subtest, testing verbal recent memory (immediate and delayed recall of story [9] (2) Boston Naming Test, testing picture recognition and word retrieval [10] (3) Rey – Osterreich’s Complex Figure (RCF) test, testing recent visual memory (copy, immediate recall, and delayed recall [11] (4) Rey Auditory Verbal Learning Test (RAVLT) testing learning abilities [12] (5) Controlled Oral Word Association Test (COWA), testing word fluency and speed of executive functions [13] (6) Trail Making Test, Parts A and B, testing visuospatial performance, executive function, and psychomotor speed [14] (7) Stroop Color Task and Stroop Color-Word test [15] testing ability to inhibit the first response, cognitive flexibility, and intellectual speed and (9) Grooved Pegboard Test (*Psychological Assessment Resources Inc, Lutz, FLA*), testing fine motor speed.

Missing test scores were generated due to patient refusal to do the test.

#### 2.3.1. *Assessment of cognitive test scores*

Variation of patients’ scores from normal (in standard deviations, SD) was based on published norms from healthy adults for WMS-R, Rey Complex Figure test, RAVLT, Trail Making test, and Stroop Color-Word tests. Performance was considered definitely abnormal (Definite Deficit – DD) for scores more than 2 SD below the mean; borderline abnormal (Borderline deficit – BD) for scores 1-2 SD below the mean; and normal for scores higher than 1 SD below the mean for the patient’s age, sex and education level.

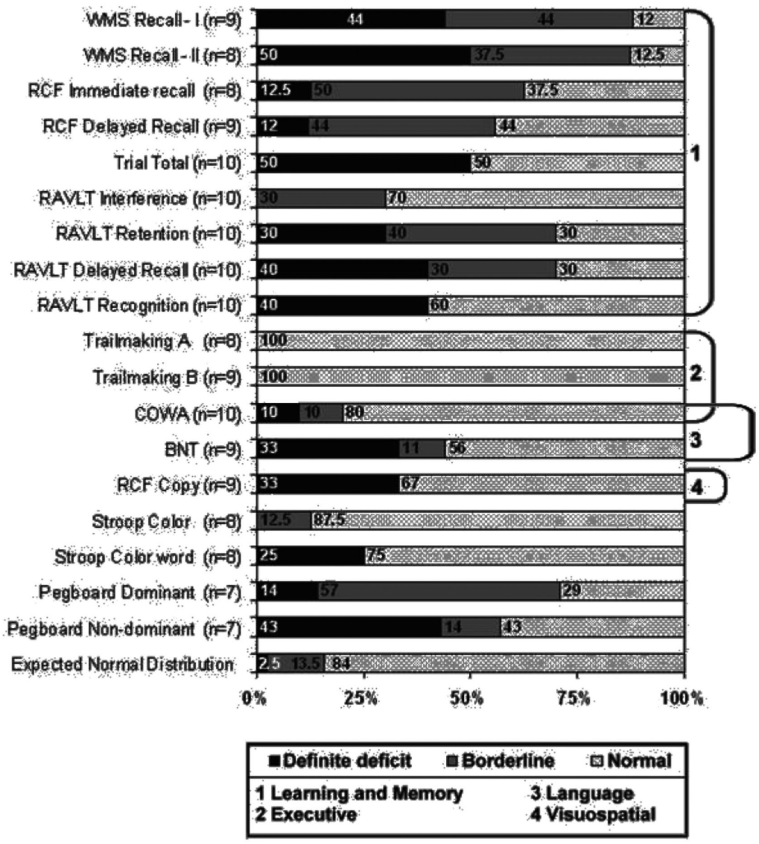

Fig. 1. Cognitive testing results (total number of patients = 10).

*2.3.2. Assessment of cognitive domain impairment*

We analyzed 4 cognitive domains:

1. Memory: 1) WMS logical memory 2) WMS delayed recall; 3) RCF immediate recall 4) RCF delayed recall; 5) RALVT
2. Executive: 1) Trail-making Test A; 2) Trail-making Test B; and 3) COWA.
3. Language: 1) BNT and 2) COWA
4. Visuospatial: 1) RCF copy

In each cognitive domain, impairment was defined as a single test score more than 2 SD below the mean, or $>$ 50% of tests more than 1 SD below the mean for age, sex and education. Any test for which the data was missing was assumed to be normal; so that the extent of cognitive domain impairment was not overestimated.

## 2.4. Statistical analysis

Statistical analysis was done using Stata 8 (StataCorp, College station, TX). The non-parametric fisher exact test was used for comparisons of prevalence between small groups.

## 2.5. Institutional Review Board approval

This study was approved by the Johns Hopkins Medical Institutions' Institutional Review Board.

## 2.6. Results and discussion

Our study indicates that patients with PTCS can have significant impairment in cognition, and this impairment was more common in memory and learning. In our study 5(50%) patients had one or more complaints of cognitive symptoms (Table 3). All of these had memory impairment besides impairment of visual-spatial skills in 3(60%) patients, executive skills in 1(20%) patient, language skill 1(20%) patient. However, even in the remaining 5 patients without any complaints related to cognitive function, 3(60%) patients had impaired memory, although none of the patients showed visulospatial deficits. MMSE scores were available in 5 of the 10 patients and ranged from 28 to 30, thus falsely indicating absence of cognitive impairment. The MMSE, which is a routinely used screening tool for dementia,

Table 2
Frequency of classically described symptoms of Idiopathic Intracranial Hypertension

| Symptom | Number (%) of patients reporting the symptom ($n = 10$) |
|---|---|
| 1. Headache | 9 (90%) |
| 2. Visual blurring | 5 (50%) |
| 3. Tinnitus | 2 (18%)* |
| 4. Dizziness/vertigo | 3 (30%) |
| 5. Gait problems | 2 (20%) |
| 6. Fainting | 1 (10%) |
| 7. Diplopia | 0 (0%) |

*1 person reported pulsatile tinnitus.

can easily miss the cognitive impairment in PTCS patients. Although, Grigoletto et al. recommended use of age, sex and education level adjusted cutoffs which have may provided high sensitivity and specificity to MMSE (sensitivity (85%) specificity (85%)) [16]. Furthermore, on the extensive cognitive testing with the neuropsychological test battery we found impairment was most commonly seen and was most severe in the verbal memory tests (WMS immediate recall, WMS delayed recall, RAVLT delayed recall, and RAVLT retention). Non-verbal memory was also found to be impaired (RCF immediate recall and RCF delayed recall); however the impairment was not as severe as that in verbal memory.

The present report is the largest study that assesses a wide range of cognitive functions in PTCS. Only few investigation have evaluated cognitive function in patients with PTCS using standardized cognitive tests [3, 5]. In a group of 5 PTCS patients who were cognitively tested because of persistent problems with "concentration, learning and memory", Sorensen et al. (35) reported that all 5 patients were impaired on verbal tests, and 3/5 patients were impaired in the non-verbal tests for cognition. The etiology of chronic intracranial hypertension was unknown in these patients. Arseni et al. assessed verbal memory by WMS testing in 85 patients with PTCS, and found that it was impaired in 20/85 (24%) patients [3]. Our study confirms the impairment in verbal memory noted earlier by Arseni et al. and Sorensen et al. It also demonstrates that some patients have impairment in non-verbal memory as well, although it is less marked.

The functional disability caused by PTC can be significant, and cognitive impairment could be an important cause of this disability in some patients [17]. A substantial proportion of our patients complained of problems with normal functioning "Table 2". However, standardized disability scores were not available for this group of patients, and further studies need to be done to clarify the relationship between cognitive impairment and functional disability.

In our study 3/10 (30%) patients had evidence of venous outflow obstruction. Similar to our study, Johnston et al. (13) found that 27/87 (31%) Pseudotumor Cerebri (PTC) patients had intrinsic occlusion of the intracranial sinuses. Others such as King et al. reported that intracranial venous sinus pressures were increased in 9/11 (82%) PTCS patients [18] and Karahilos et al. (16) reported elevated superior sagittal sinus or sigmoid sinus pressure in 8/8 (100%) patients in whom manometry was performed, even though only 3/8 (37%) patients had an abnormal venogram. It has therefore been proposed that intracranial venous pressure is the common final pathway for all etiologies of PTCS [19], Impaired outflow from intracranial venous sinuses in PTCS patients is one possible explanation for the cognitive impairment seen in our patients. Cognitive impairment can occur in patients with confirmed Cerebral Venous Sinus Thrombosis (CVST) [20,21]. Bruijn et al. found cognitive impairment in 16/59 (27%) of CVST patients, while Buccino et al described impairment in 7/38 (18%) of CVST patients [20,21].

However, it is difficult to estimate what proportion of patients with CVST develop cognitive impairment, since both studies conducted cognitive testing at least a year after CVST was diagnosed and in most cases, treated with anticoagulation and/or heparin. There are no studies involving detailed cognitive testing in the early stage of the disease [21].

While presence of venous sinus outflow obstruction in 30% of our population would have contributed to the cognitive impairment in these patients, studies done in pre-MR venography era [3,5] could not have evaluated patients for venous obstruction.

## 3. Limitations

This study is limited by few numbers of patients and absence of controls. Therefore, results of this pilot study should be cautiously interpreted considering that anxiety/depression or chronic headaches may partly account for cognitive complaints in PTCS patients and confound the results [22]. Furthermore, population of patients with PTCS is very heterogeneous [2, 23] and deficits may differ between presentations and etiologies. Thus, inclusion of appropriate number of matched controls in future studies is necessary to define cognitive deficits in PTCS. Another limitation to this

Table 3
Cognitive evaluation results by complaints

| Complaints | N | Memory impairment (%) | Executive skills impairment (%) | Language skill impairment (%) | Visuospacial skill impairment |
|---|---|---|---|---|---|
| Any Cognitive Complaints | 5 (50%) | 5 (100) | 1 (20) | 1 (20) | 3 (60) |
| Recent memory | 5 (50%) | 5 (100) | 1 (20) | 1 (20) | 3 (60) |
| Long term memory | 2 (20%) | 2 (100) | 0 (0) | 0 (0) | 1 (50) |
| Problems balancing bills or organizing tasks | 3 (30%) | 3 (100) | 1 (33.34) | 1 (33.34) | 2 (66.67) |
| No Cognition related complaints | 5 (50%) | 3 (60) | 0 (0) | 2 (40) | 0 (0) |

study is that patients were not evaluated for reversal of cognitive deficits following treatment. Post-treatment follow-up evaluation of cognitive function is needed to confirm causal association between PTCS and cognitive disturbances.

## 4. Conclusion

Our results indicate that patients with PTCS can have significant cognitive impairment, particular in learning and memory. The prevalence of cognitive impairment should be studied in a larger cohort. The relationship of cognitive impairment with chronically elevated intracranial pressures and its role in contributing towards patient morbidity in PTCS needs to be investigated further. Further studies are required to determine whether shunt surgery improves cognition in PTCS patients.

## Acknowledgements

This work was supported by a grant from the Salisbury Foundation, Medtronic Inc. and by the Schoendorf foundation.

We thank Sumit Kapoor at the Johns Hopkins University Hydrocephalus Research Center for his kind help and review of this manuscript.

## Financial disclosure

The Adult Hydrocephalus Program at Johns Hopkins is supported by the Salisbury Foundation, Medtronic and the Schoendorf Foundation. Dr. Rigamonti and Dr. Williams have received honoraria from Medtronic and Codman to speak about hydrocephalus. Part of Dr. Kharkar's salary was paid by a grant from Medtronic during the study period.
